# Biometric and genetic differences in kelabau ( *Osteochilus *spp.) as revealed using cytochrome c oxidase subunit 1

**DOI:** 10.12688/f1000research.17319.1

**Published:** 2019-02-12

**Authors:** Nur Asiah, Junianto Junianto, Ayi Yustiati, Sukendi Sukendi, Melta Rini Fahmi, Zainal A. Muchlisin, Muhamad Kadapi

**Affiliations:** 1 Department of Fisheries and Marine Science, Padjadjaran University, Sumedang, West Java, 45363 Sumedang, Indonesia; 2 Aquaculture Department, Riau University, Pekanbaru, Riau, 28293, Indonesia; 3 Aquaculture Department, Research Center for Ornamental Fish Culture, Depok, West Java, 16436, Indonesia; 4 Aquaculture Department, Syiah Kuala University, Banda Aceh, Aceh Darussalam, 23111, Indonesia; 5 Agronomy Department, Padjadjaran University, Sumedang, West Java, 45363, Indonesia

**Keywords:** DNA barcoding, Kelabau Fish, Common Rivers of Riau, Population Structutre

## Abstract

**Background:** Kelabau ( *Osteochilus* spp.) is a freshwater fish commonly found in the rivers of Riau, Indonesia. Researchers believe that these are *Osteochilus kelabau*; however, accurate taxonomic determination of these fish in Riau waters has not been made. The purpose of this study was to facilitate the identification of the kelabau based on its morphology and genetics using biometric and cytochrome c oxidase subunit 1 ( *CO1*) analyses, respectively.

**Methods:** Fish samples were collected from the Siak, Kampar and Rokan rivers in Riau Province, Indonesia. The DNA of 90 fish was extracted from the caudal fins using a DNA extraction kit, after which it was amplified using primers Fish-F1 and Fish-R1. Sequencing was conducted by Applied Biosystems Macrogen Korea, and the DNA sequences were then edited and aligned using MEGA v. 7. All samples were BLAST-searched for identification using the National Center for Biotechnology Information and BOLD System. Phylogenetic trees were constructed, and similarity index was calculated using accession numbers AP011385.1 and KC631202.1 in GenBank.

**Results:** Analysis of the consensus barcode sequence for 86 species revealed a high percentage of barcode matches (96%–97% in GenBank and 96.6%–96.76% in the BOLD System). The nucleotide distance between groups of kelabau from the different rivers based on the Kimura 2-parameter model gave the following results: 0.05% between groups from the Siak and Kampar rivers, 0.09% between those from the Siak and Rokan rivers and 0.05% between those from the Kampar and Rokan rivers. The nucleotide distance between the groups in the Siak (0.09%), Kampar (0.00%) and Rokan (0.10%) Rivers indicated that the kelabau in those rivers were related to each other.

**Conclusions:** Based on the results of the research data using *CO1* and biometric analyses, the kelabau were confirmed to be *O. melanopleurus.*

## Introduction

Kelabau are ancient fish belonging to genus *Osteochilus* of family Cyprinidae. The species is distributed throughout Thailand, Vietnam, Peninsular Malaysia, Borneo and Sumatra ^1, 2^. In Sumatra Island in Indonesia, the fish is commonly found in the Siak, Kampar and Rokan rivers in Riau Province ^3, 4^. The demand for it as a food resource is increasing; however, its populations are decreasing in several major rivers in Sumatra, particularly in Riau Province ^5, 6^, and its cost is increasing. Several factors, such as overfishing, electrofishing and changes in river ecosystems, might play a role in the decline of the populations of these as well as many other fish ^5, 7^.

According to local fishers in Riau, kelabau are divided into two types on the basis of morphology; however, there is no detailed information about these fish types. Thus, a study was needed to identify the species using morphological and molecular methods to determine these types in the Siak, Rokan and Kampar rivers. Identification of any species using morphological traits can be difficult and can lead to errors ^8^; therefore, owing to morphological similarities among *Osteochilus* spp., molecular markers, such as DNA barcodes, are important to identify the fish species uses a specific sequence region (i.e. cytochrome c oxidase subunit 1 ( *CO1*)) to identify a species and is a technique that can identify taxonomic units as well as biodiversity for determining species of several organisms ^8– 12^. Unlike molecular phylogeny used to determine relationships among species, the purpose of DNA barcoding is to identify unknown or undetermined species into phylogeny ^13^. The common mitochondrial (mt) DNA region used as a barcode in protists and animals comprises 600 bp. In addition, *CO1* is one of the genetic markers used to identify insects, birds, primates and fish to species ^14– 16^. MtDNA *CO1* is selected as a target in DNA barcoding because it is a highly conserved site. This method has advantages over the morphological identification approach in that it is fast, reliable and it can be used for all types of samples because it uses a single gene along with mutations in the nucleotides to acknowledge the taxonomic features of each species ^16^.

The study on DNA barcoding for freshwater fish has been widely practised in various countries, including Nigeria ^17^, Malaysia ^18^, Philippines ^19^, Canada ^12^ and Indonesia ^8, 20– 22^. The method has been successfully validated for the taxonomic status within *Rasbora* in Lake Laut Tawar ^23^; Anguillidae in Aceh waters ^24^; Ornamental fish from Peat lands ^8^; Channidae in Peninsular Malaysia, Sarawak, Sumatera, Borneo, Myanmar, Vietnam, India, Germany, Singapore and the United Kingdom ^25^ and Cichlidae in northeastern Nigeria ^26^; therefore, it can be used to equally successfully validate the taxonomic unit of the kelabau using its morphology supported by molecular data. This information is crucial for designing a remedial course of action with regard to the conservation strategy for this species in the Siak, Kampar and Rokan rivers in Riau Province, Indonesia.

## Methods

### Ethics

The study population was collected and sampled according to the guidelines on the use of living organisms for research from the Laboratory of the Faculty of Fisheries and Marine, Riau University, Indonesia.

### Sampling sites and collection

**Figure 1.**
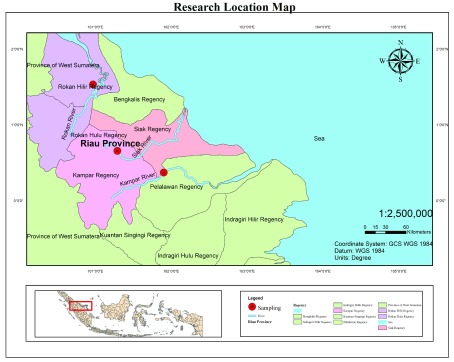
Sample sites for *Osteochilus melanopleurus* from the Siak (N: 00°39'22.28'' and E: 101°17'28.67''), Kampar (N: 00°22'13.64'' and E: 101°54'11.97'') and Rokan rivers (N: 01°22'33.65'' and E: 100°58'26.76''), Riau Province, Indonesia.

A total of 90 kelabau (30 fish from each river) were collected from the Siak, Kampar and Rokan rivers ( Figure 1). Fish were caught using a gill net 3 m deep and 20 m long with a 12.7-cm mesh. The gill nets were installed in the river water close to the riverbank and remained for 24 h from 08:00 to 08:00 the following day. The fish collected were counted using handcounter and cleaned using freshwater. A number of 50-mm caudal fin tissue samples were taken using a sterile scissors and preserved in ethanol, after which a photo of each fish sample was taken for documentation using a digital camera.

All samples were preserved in 3-kg sample bags which were labelled according to site location, date and serial number. Before preservation, the fish samples were injected with 10% formalin. The fish samples were then transported to the laboratory for further evaluation. The morphologies of the collected fish were identified up to species level using the identification book produced by the Indonesian Institute of Sciences ichthyology museum ^2, 27^. The fish morphologies observed were length, colour, shape of scales, mouth shape, barbels, number of fins and special marks on the body.

### Biometrics

Biometric analyses were used to measure morphological characteristics in this study ^2^. This tool is considered conventional for identifying organisms. Molecular identification using *CO1* gene sequences has been supported for providing additional organism classification.

### DNA isolation and amplification

DNA was extracted using the spin-column method from the gSYNC DNA Extrusion Kit (Geneaid Catalogue No. GS 300, Taiwan). The extracted DNA was than transferred to a 1X Tris–borate ethylenediaminetetraacetic acid (TBE) solution with a 1.5% agarose gel and Pegreen gel dye (PEQLAB Biotechnologie GmbH, Erlangen, Germany) ^22^. The quantity of DNA was visualised with the help of a GeneQuant Spectrophotometer by adding 78 μL nuclease-free water in a cuvette along with 2 μL DNA. The DNA was then amplified using the universal primer Fish-F1 (5'-TCA-ACC-AAC-CAC-AAA-GAC-ATT-GGC-AC-3') and Fish-R1 (5'-TAG-ACT-TCT-GGG-TGG-CCA-AAG-AAT-CA-3') with a target of 707 bp and 655 bp ^28^, respectively. The amplification thermocycling conditions as follow: the PCR condition using pra PCR (94°C for 5 min), 35 cycles of denaturation (94°C for 30 s), annealing (56.6°C for 30 s) and extension (72°C for 30 s), followed by post-PCR extension (72°C for 5 min) and hold (4°C for 5 min). PCR results were analysed using 1.5% agarose gel at 100 V to assess the bands, and only the clear products were sent to Applied Biosystems Macrogen Korea for sequencing.

### Controlling molecular samples and sequence quality

The PCR amplicon was 707 bp, which implied that no sequence of DNA was derived from mtDNA nuclear mitochondrial DNA segments (NUMTs), because a NUMT barely reaches 600 bp ^12^. The selected *CO1* sequences were entered into GenBank and the BOLD System databases to compare the alignment of nucleotide sequences and 99%–100% values with that with no insetions/deletions. All sequences were aligned using ClaustalW with MEGA v.7 ^29^.

## Data analysis

### Blasting of *CO1* by NCBI (GenBank) and BOLD System (online)

The entire nucleotide sequence obtained from the sequence chromatogram was assembled using DNA Baser Assembler, aligned and then analysed using MEGA 7. It was further aligned (multiple alignments) using the reference NCBI GenBank accession numbers AP011385.1 and KC631202.1. Similarly, the percentage of *CO1* sequences were blasted using NCBI Blast and BOLD Systems databases.

### Nucleotide variations

Nucleotide variations among samples were analysed using dnaSP v.5 ^30^. The parameters of these calculations were haplotype number, variable site, parsimony site, haplotype diversity and nucleotide diversity.

### Phylogenetic tree

Phylogenetic trees were estimated using all samples from the three populations and calculated according to the Tamura-Nei model ^31^ using MEGA 7 ^29^.

### Nucleotide distance

The distance among the nucleotide bases of the mtDNA *CO1s* was analysed using the Kimura 2-parameter model ^32^. The nucleotide distances between and within the populations were examined according to the model based on the similarity of frequencies and ratios of transition to transversion (Ti:Tv) using MEGA 7 ^29^.

## Results

### Morphological identification

**Figure 2.**
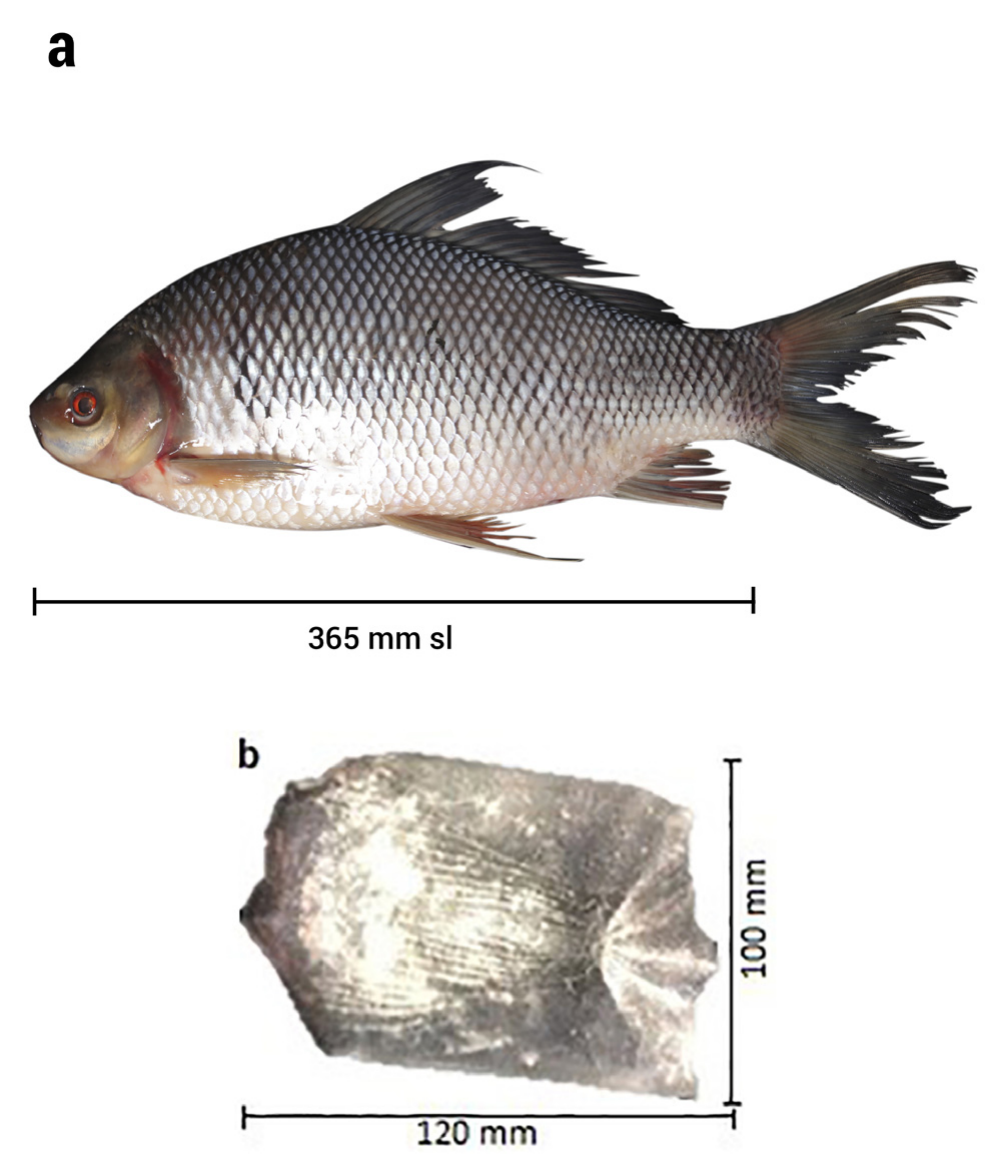
( **a**) Kelabau ( *O. melanopleurus*) and ( **b**) ctenoid scale of *O. melanopleurus*.

The morphological traits of all kelabau used in this study matched those of *O. melanopleurus*. We used the important morphological traits to identify these fish according to Kottelat *et al*. ^2^. The morphological characteristics measurement of *O. melanopleurus* showed that the fish have 16–19 branched dorsal rays, the number of scales was ranged from 10.5 to12.5 in between dorsal origin and lateral line, the number of circum peduncular rows of scale was ranged from 20 to 24 and lips covered with folds and plicae and there was no hard tubule at the tip of the mouth ( Figure 2a). This species has one pairs of barbels at above and one pairs at bottom, dark hazy blotches near above of the pectoral fins. The body is brownish, with the bottom brighter than the top and the type of ctenoid scales ( Figure 2b). Raw biometric data are available on OSF ^33^.

### Genetic analysis

**Figure 3.**
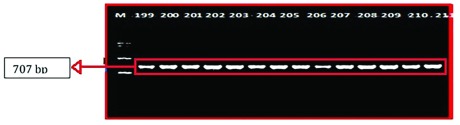
DNA amplified using Fish-F1 and Fish-R1 primers; M= Marker 100 bp (Vivantis, Malaysia); 199, 200, 201, 202, 203, 204, 205, 206, 207, 208, 209 and 210= Rokan River; 211= Kampar River.

A sequence amplified by Fish-F1 primer was successfully identified in 86 of 90 samples of mtDNA fish. The base length of the *CO1* nucleotide obtained from the formulation process and electrophoresis ( Figure 3) was ~707 bp.

Based on genetic analysis using the Tamura-Nei model, there was an unequal distribution of all nucleotides with the following frequencies: adenine (A), 26.73%; thymine (T), 30.44%; cytosine (C), 25.93% and guanine (G), 16.90% ( Table 1). The rates ratio between transition and transversion was 10.257 purines and 1.915 pyrimidines, and the overall transition and transversion bias was R= 2.499 based on Tamura-Nei model ^34^. The pattern of nucleotide distribution of A, T, C and G was T > A > C > G, but the carps which cultivated in India have the following pattern of nucleotide distribution: T > C > A > G ^35^.

**Table 1.** Maximum composite likelihood estimates of the pattern of nucleotida substitution.

	A	T	C	G
**A**	-	*4.03*	*3.34*	**22.95**
**T**	*3.54*	**-**	**6.57**	*2.24*
**C**	*3.54*	**7.72**	-	*2.24*
**G**	**36.29**	*4.03*	*3.34*	*-*

Bold: different transitional subtitutions; italic: tranversional subtitutions.

The nucleotide distances between nucleotide bases within the groups indicated that the values of the nucleotide base sequences within the fish population were 0.0009, 0.0000 and 0.0010 in the Siak, Kampar and Rokan rivers, respectively. The evolutionary distance between the nucleotides of the groups had a comparative difference in the nucleotide sequences of 0.0005 for fish from the Siak and Kampar rivers, of 0.0009 in those from the Siak and Rokan rivers and of 0.0005 for those from the Rokan and Kampar rivers. Based on the nucleotide distance, the fish were identified as being from the same species (0.06%) ( Table 2).

**Table 2.** Nucleotide distances among the populations.

	1	2	3	4
**Siak**	-			
**Kampar**	0.0005	-		
**Rokan**	0.0009	0.0005	-	
**GenBank (AP011385.1)**	0.0367	0.0366	0.0368	-
**GenBank (KC631202.1)**	0.0351	0.0350	0.0351	0.0048

Source: Kimura estimation of 1980: “Evolutionary Divergence over Sequence Pair Between Groups” ^32^.

*CO1* had 612 conserved sites (98%), 9 variable sites (1.45%), 4 informative parsimony (0.64%) and 5 singleton sites (0.81%). The highest nucleotide variation was in the Rokan river population (0.00100 ± 0.00032); whereas, the Kampar river population had no nucleotide variation based on DnaSP5 calculations ( Table 3). Using the NCBI database with accession numbers AP011385.1 and KC631202.1, the DNA sequence of Kelabau was identified as belonging to *O. melanopleurus* with 96%–97% accuracy, query coverage of 99%–100% and an E-value of 0.0. While based on the BOLD System, the identity of all samples was 96.60%–96.93% accurate.

**Table 3.** Nucleotide variation in mtDNA *CO1* of *Osteochilus melanopleurus* by DnaSP5.

Sampling location	Number of sites	Number of sequences	Haplotype number	Variable site	Parsimony site	Haplotype diversity	Nucleotide diversity
**Siak**	621	28	6	6	2	0.439 ± 0.114	0.00090 ± 0.00029
**Kampar**	621	30	1	0	0	0	0
**Rokan**	621	28	6	6	2	0.437 ± 0.113	0.00100 ± 0.00032

Source: Nei, 1987 for haplotype and nucleotide diversity ^36^.

**Figure 4.**
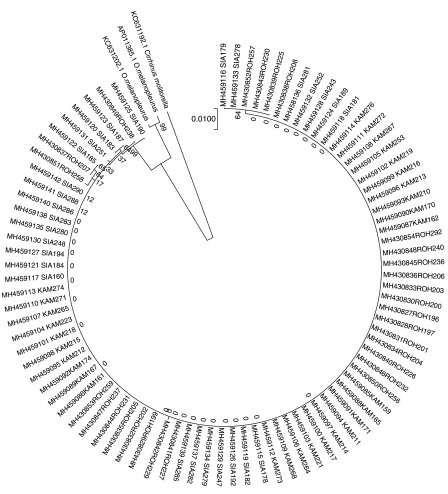
Phylogenetic tree of kelabau ( *Osteochilus melanopleurus*) based on the neighbour-joining model.

In the phylogenetic tree consisted of two major groups ( Figure 4). The first group was *Cirrhinus moltonela* (GenBank KC631192.1) and it was divided from *O. melanopleurus*. The second group was differentiated into two sub groups, *O. melanopleurus* from GenBank (AP011385.1 and C631202.1) and 86 fish samples from Kampar, Siak and Rokan rivers. The 86 samples have BLASTN similarity values with *O. melanopleurus* of 96%–97%.

## Discussion

Overall, the morphological traits and DNA barcoding showed that the majority of, if not all, kelabau fish in the three rivers at Riau Province were *O. melanopleurus*. Although the local fishermen have reported that there are two types of kelabau fish in the Riau river, these differences could the result of environmental changes and overfishing.

Environmental changes can cause fish death or migration to suitable habitats. Overfishing using both legal and illegal methods has also triggered the decline of certain species ^5, 7^. In addition, our results suggested that there was little nucleotide diversity among *O. melanopleurus* in the Siak, Rokan and Kampar rivers in Riau Province, particularly the fish in the Kampar river

The lack nucleotide diversity of *O. melanopleurus* from the three rivers was likely to be caused by limited opportunities for kelabau migration, so that the genetic exchanges with other populations are very small ^7^; morevoer, the lack nucleotide diversity is believed to be caused by inbreeding, and overfishing ^8, 37^.

In addition, Kelabau from the Siak and Rokan rivers were designated as one sub-sub group in group two ( Figure 4) because both rivers are geographically connected, allowing for hybridisation, whereas, there is inbreeding of these fish in the Kampar River, which causes a nucleotide diversity value of 0.0. The same deductions were drawn from previous studies on *Desmopuntius pentazona* and *D. rombochelatus*, although the taxonomies of the two fish are different. Nevertheless, based on a genetic difference of only 0.4%, the two species were grouped into one cluster ^8^. These are distributed throughout Asia, the Mekong and Chao Praya river basins, Peninsular Malaysia, Sumatra and Borneo ^1, 2^.

The identity of a species was derived using the morphological identification method to distinguish between species or individuals ^38, 39^. Basically, the genetic identification of a species can be done using mtDNA *CO1*, a more effective approach than using rRNA ^8, 35^. The nucleotide locus and mutations were used as references to conduct DNA barcoding in all fish samples ^16^. Previous studies have identified several species using DNA barcoding, such as ornamental fish of wetlands ^8^, wetland fish larvae ^39^, rainbow fish ^40^, Cyprinidae fish ^41^, salmon and trout ^10^ and freshwater fish ^12, 22^. Furthermore, the phylogenetics of *CO1* sequences can effectively show congeneric and confamilial species ^12^.

The phylogenetic trees cauld describe the line of biological evolution from species or organisms with a different ancestry ^42^. Nonetheless, the results of all these species did not show a 100% undistinguishable identity. The branch length between species leading to a gap in the pairwise distance distribution is referred to as the barcoding gap in *CO1* ^26^. Intra-species relationships were quite high in all samples, which confirmed that kelabau ( *O. melanopleurus*) were native in the three rivers and had the ability to adapt to changes in environmental conditions ^37^.

Moreover, the existence of inter-nucleotide patterns and distances between A, T, C and G in the chromosomes showed the characteristics and genetic signs that distinguished each of the individuals, even though they belong to the same species ^43^. This is reinforced by referencing the phylogenetic tree made using the neighbour-joining model.

The identification of fish species is normally conducted using morphological characteristics such as dorsal fins, pelvic fins, pectoral fins, anal fins, linea lateral fins, upper linea lateral fins, lower linea lateral fins, around body fins, and caudal peduncle fins; however, in this study, we used 12 morphological traits as described by the classification system of Kottelat *et al.* ^2^. These results supported the classification using biometric data that all fish in the three rivers were *O. melanopleurus*. The morphological characteristics were consistent with the species having a relatively large body with a standard length of 119–560 mm, lips covered with folds and plicae, no tubercles on the snout, a pair of maxillary barbels, and a pair of lower jaw barbels. The body is brownish, with the bottom brighter than the top. Dark hazy blotches near above of the pectoral fins, which is a special trait of *O. melanopleurus.*

However, this method can be difficult, and molecular identification is necessary. In particular, using mtDNA *CO1* was an effective approach ^12, 20^. The results from nucleotide distance data based on the Kimura 2-parameter model indicated that the nucleotide distance among the fish was short in intraspecific species using mtDNA *CO1* ^37^, which was supported by data showing that the percentage identity in *O. melanopleurus* species ranged between 96% and 97%. The Kelabau fish from the three sample sites were identified as *O. melanopleurus* by percentage identity, supported by an E-value of 0.0 and a 99%–100% query cover. The p-value indicated that the BLASTN results contained no errors. In addition, the low nucleotide distance values (<3%–5%) among the samples of *O. melanopleurus* from the Siak, Kampar and Rokan rivers, indicated that all samples were monophyletic.

## Conclusion

Based on our findings, we concluded that 86 of the 90 samples of kelabau from the Siak, Kampar and Rokan rivers in Riau were *O. melanopleurus*, as revealed by their morphological traits and the molecular analyses.

## Data availability

### Underlying data

*CO1* gene sequences and raw biometric data of *Osteochilus melanopleurus* from Riau rivers can be found on OSF.

*CO1* gene sequence DOI: https://doi.org/10.17605/OSF.IO/XGEZD ^44^.

Raw biometric data DOI: https://doi.org/10.17605/OSF.IO/CFGM8 ^33^.

Data are available under the terms of the Creative Commons Zero "No rights reserved" data waiver (CC0 1.0 Public domain dedication).

The raw *CO1* sequence data were also deposited in GenBank and can be found under sequential accession numbers MH430827-MH430854 and MH459085-MH459142.
