# Supplementary material for: Biometric and genetic differences in kelabau ( Osteochilus spp.) as revealed using cytochrome c oxidase subunit 1
Source: F1000Res. 2020 Mar 10;8:177. Originally published 2019 Feb 12. [Version 3] doi: 10.12688/f1000research.17319.3 (PMC7008605; doi:10.12688/f1000research.17319.3)
Supplement: Peer review history

# Peer review: Reviewer response for version 3

**DOI:** 10.5256/f1000research.24696.r61112

**Published:** 2020-03-12

**Authors:** Ja’afar Nuhu Ja’afar

**Affiliations:** 1 Department of Biotechnology, Modibbo Adama University of Technology (MAUTECH), Yola, Nigeria; 2 Chevron Biotechnology Center, Modibbo Adama University of Technology (MAUTECH), Yola, Nigeria

Please find my highlighted copy of the manuscript here. The areas highlighted in the manuscript are supposed to portray the integrity and diligence of both the journal and reviewers, thus, maintaining scientific standards. Aside from this, the manuscript is ready to be indexed.

I confirm that I have read this submission and believe that I have an appropriate level of expertise to confirm that it is of an acceptable scientific standard.

# Peer review: Reviewer response for version 2

**DOI:** 10.5256/f1000research.24129.r59013

**Published:** 2020-02-10

**Authors:** Indra Junaidi Zakaria

**Affiliations:** 1 Departement of Biology, Faculty of Math and Science, Andalas University, Padang, Indonesia

I confirm that I have read this submission and believe that I have an appropriate level of expertise to confirm that it is of an acceptable scientific standard.

# Response

**Published:** 2020-02-08

**Authors:** Nur Asiah

**Affiliations:** Riau University, Indonesia

Dear Mr. Indra Junaidi Zakaria,

Thank you for your suggestions on our manuscript. Through this letter I would like to explain the revision of my article titled *“Biometric and genetic differences in kalabau (Osteochillus spp) as revealed using Cytochrome C Oxidase subunit 1”.* I have revised these following points:

- Grammatical and spelling mistakes throughout the paper have been revised.
- The introduction has been improved by adding information on the difference of the characteristics of two species of *kelabau *fish *(Osteochillus spp)*. As there are 2 types of fish that are named as “kelabau fish” by local people, information on the difference of those fishes was obtained by interviewing local fishermen. The presence of this information may be useful to give more explanation about the difference and the similarity of *kelabau* fish present in Riau in general.
- A phrase *“The demand for it as …” until “… the population of these as well as many other fish”* has been deleted as it is not correlate with the theme of this manuscript.
- Finally, the author has also added a quote in the following paragraph: *“The study population was collected and sampled …” until “… of the Faculty of Fisheries and Marine Science, Riau University, Indonesia”*.
- The discussion section has been improved by comparing the result of this study with more articles available.

Thank you for your suggestions and we hope that our article will be approved.

Best Regards,

Nur Asiah and the authors

# Peer review: Reviewer response for version 2

**DOI:** 10.5256/f1000research.24129.r59015

**Published:** 2020-02-07

**Authors:** Pallipuram Jayasankar

**Affiliations:** 1 Fish Genetics and Biotechnology Division, ICAR-Central Institute of Freshwater Aquaculture, Bhubaneswar, Odisha, India

I have gone through the revision/corrections effected by the authors in the manuscript “Biometric and genetic differences in kelabau ( *Osteochilus *spp.) as revealed using cytochrome c oxidase subunit 1”. The manuscript has been improved as per my suggestions. I recommend to consider its indexing.

I confirm that I have read this submission and believe that I have an appropriate level of expertise to confirm that it is of an acceptable scientific standard.

# Response

**Published:** 2020-02-08

**Authors:** Nur Asiah

**Affiliations:** Riau University, Indonesia

Dear Mr. Pallipuram Jayasankar,

 Thank you very much for your approved on our article, and thank you for your suggestions for the good of our article.

 Best regards,

 Nur Asiah and the authors

# Peer review: Reviewer response for version 2

**DOI:** 10.5256/f1000research.24129.r59014

**Published:** 2020-02-03

**Authors:** Ja’afar Nuhu Ja’afar

**Affiliations:** 1 Department of Biotechnology, Modibbo Adama University of Technology (MAUTECH), Yola, Nigeria; 2 Chevron Biotechnology Center, Modibbo Adama University of Technology (MAUTECH), Yola, Nigeria

In version 1 of the manuscript, I have highlighted some sections that may require the authors' attention. However, none of those have been effected. Perhaps, the authors did not get the script I have corrected. It is available if needed.

I confirm that I have read this submission and believe that I have an appropriate level of expertise to confirm that it is of an acceptable scientific standard, however I have significant reservations, as outlined above.

# Response

**Published:** 2020-02-08

**Authors:** Nur Asiah

**Affiliations:** Riau University, Indonesia

Dear Mr. Ja'afar Nuhu Ja'afar,,

Thank you for your suggestions on our manuscript and we apologize for the late to response your suggestion because we need to discuss with other authors. Here we would like to inform that we already revised paragraph three by adding reasons for limited variation such as inbreeding within the species due to limited migration and we also added discussion about data on table 3.

Variation in nucleotide diversity of the fish from the sampling areas are different. In the Kampar River, the fish was sampled from the relatively narrow area and it may cause the lowness of the nucleotide diversity. The diversity was 0 and it means that the nucleotide of the fish samples was almost identical. Freeland *et al*., (2011) stated that the low nucleotide diversity may occur as the habitat of the fish is very suitable and the fish may not face environmental related problem that trigger any genetic changing. However, the nucleotide diversity of the fish from the Siak and Rokan River, was higher than Kampar River. Siak and Rokan Rivers have relatively low water quality that was caused mainly by anthropogenic activities. Changing in water quality may trigger the fish to adapt with that environmental condition and it may cause changes in genetic variation. Freeland *et al*., (2011) stated that fish population with high genetic variation may be able to face problems related to environmental changing.

Finally, we want to thank you very much for your suggestion on our manuscript and we hope that our manuscript will be approved.

Best regards,

Nur Asiah

# Peer review: Reviewer response for version 1

**DOI:** 10.5256/f1000research.18938.r55212

**Published:** 2019-11-04

**Authors:** Pallipuram Jayasankar

**Affiliations:** 1 Fish Genetics and Biotechnology Division, ICAR-Central Institute of Freshwater Aquaculture, Bhubaneswar, Odisha, India

DNA barcoding is an important and useful tool to ratify species status in circumstances where morphology-based taxonomy fails or nearly-fails to perform unambiguous identification of species. The authors in the present study have taken up an important group of freshwater fish. There are many grammatical errors and some technical flaws in the manuscript. I felt one major drawback could be their total silence on the nominal species of *Osteochilus kelabu*. Of course using barcoding they have proved that the species is *O. melanopleurus*. However, they should have shown a phylogenetic comparison of these two nominal species to rule out confusion in identification. Morphological method followed does not appear to be robust. They are advised to use TRUSS MORPHOMETRICS which can be performed using user-friendly software nowadays. In the phylogenetic tree the majority of bootstrap values do not appear to be robust; hence it could reflect a spurious relationship.

I confirm that I have read this submission and believe that I have an appropriate level of expertise to confirm that it is of an acceptable scientific standard, however I have significant reservations, as outlined above.

# Peer review: Reviewer response for version 1

**DOI:** 10.5256/f1000research.18938.r53842

**Published:** 2019-10-04

**Authors:** Ja’afar Nuhu Ja’afar

**Affiliations:** 1 Department of Biotechnology, Modibbo Adama University of Technology (MAUTECH), Yola, Nigeria; 2 Chevron Biotechnology Center, Modibbo Adama University of Technology (MAUTECH), Yola, Nigeria

Generally, the article needs to be proofread by an expert. There are a lot of areas I have highlighted to guide the authors.

 In paragraph three of the discussion section, the authors should be more scientific in providing reasons for limited variation such as inbreeding within the species due to limited migration. More so, if COI is known to be conserved within a species, it is not expected to change within a small time frame of genetic event. In general, the discussion should be rewritten to explain the results and interpretations of the study.

 In addition, I suggest the authors contact an expert that can explain/interpret the results obtained using DNASP. The explanation of the results from the software should be more than presenting the values.

 The gel result will be more appreciable if the marker has been properly labelled.

I confirm that I have read this submission and believe that I have an appropriate level of expertise to confirm that it is of an acceptable scientific standard, however I have significant reservations, as outlined above.

# Peer review: Reviewer response for version 1

**DOI:** 10.5256/f1000research.18938.r44418

**Published:** 2019-03-04

**Authors:** Indra Junaidi Zakaria

**Affiliations:** 1 Departement of Biology, Faculty of Math and Science, Andalas University, Padang, Indonesia

**Generally:**

- There are still a lot of grammatical and spelling mistakes throughout the paper. Therefore, the authors should remove all of the grammatical and typographical errors as well as spelling mistakes. Use past tense and rephrase the sentences.

**Introduction:**

- Inconsistency in writing in the first paragraph, the first line: it is written as “genus” but in the second row it is written as “species”. *Kelabau* are ancient fish belonging to the genus *Osteochilus* of family Cyprinidaes. The species are distributed throughout Thailand, Borneo and Sumatra (etc.).
- According to local fishers in Riau, *kelabau* are divided into two types on the basis of morphology. Although there is no detailed information about these fish types, please, the authors should explain what the differences in the characteristics are of the two species which the fishermen know.
- Sentences from: “The demand for it as…” until “…the population of these as well as many other fish.” I suggest deleting it, because it has no correlation with the theme of this manuscript which explains the verification of the species of fish *kelabau* using cytochrome c oxidase - it is not about increasing demand and the problem of decreasing populations of *kelabau* fish.

**Methods:**

- Cite references in the following paragraph: “The study population was collected and sampled…” until “…of the Faculty of Fisheries and Marine, Riau University, Indonesia.”

**Results:**

- Ok.

**Discussion:**

- The Discussion is not up to the mark, especially in paragraphs one and two; please rewrite back with an explanation of how the characteristics of the two species of fish differ according to fishermen and the reason that these differences were caused by environmental changes and overfishing.  

I confirm that I have read this submission and believe that I have an appropriate level of expertise to confirm that it is of an acceptable scientific standard, however I have significant reservations, as outlined above.
